# Over the Counter: Self-Care or Self-Harm? A Mixed Method Study Protocol on Prevalence and Determinants of Self-Medication Practices in Wardha

**DOI:** 10.7759/cureus.65653

**Published:** 2024-07-29

**Authors:** Sana Ahmed, Sonali Choudhari, Abhay Gaidhane

**Affiliations:** 1 Community Medicine, Jawaharlal Nehru Medical College, Datta Meghe Institute of Higher Education and Research, Wardha, IND; 2 Community Medicine, School of Epidemiology and Public Health, Jawaharlal Nehru Medical College, Datta Meghe Institute of Higher Education and Research, Wardha, IND

**Keywords:** pandemic, self-prescribed drugs, healthcare delivery, prescription drugs, adverse drug reactions

## Abstract

Background

Self-medication (SM), a common practice globally, possesses a dual challenge of being a self-care strategy and a potential source of harm observed across all age groups. The study is being conducted to gauge the prevalence of SM of prescription drugs with their over-the-counter access, thus addressing the delicate balance between self-care and self-harm related to SM.

Material and methods

This ongoing convergent parallel mixed method study with quantitative and qualitative components will be conducted on a sample size of 180 subjects aged more than 18 years from an urban community. For the quantitative component, a semi-structured questionnaire will assess the prevalence of SM, types of medications used, reasons for self-medicating, and socio-demographic factors influencing these practices. Concurrently, qualitative interviews delve deeper into the beliefs shaping SM practices. Sampling will be purposive to capture diverse perspectives, with data analyzed using statistical tools.

Results

This study protocol will offer a comprehensive understanding of the prevalence and determinants of SM practices. The quantitative data provide numerical insights into SM trends, while the qualitative findings elucidate the nuanced factors driving individuals' SM choices.

Conclusions

A multifaceted view of SM practices will be provided, aiding in developing interventions to promote safe and effective self-care while mitigating the risks of self-harm through SM. Anticipated findings can include a widespread prevalence of SM amongst the general urban populace. Significant associations can also be expected to be found with various independent variables. The results will be instrumental in informing public health policies and healthcare practices toward enhancing patient safety and well-being.

## Introduction

Self-medication (SM) is described as the acquisition and ingestion of one or more drugs without consulting a medically qualified physician for diagnosis, treatment, or prescription oversight. It encompasses the use of non-prescription medications by individuals without professional guidance. The wide accessibility of diverse drugs and insufficient and unequal healthcare services contribute to a rise in SM practices in developing nations such as India [1]. Studies carried out in India have observed SM prevalence rates ranging from 29.1% to 55%, varying based on study methodologies and geographical locations [2,3]. Previous studies conducted both domestically and Internationally have identified numerous factors, ranging from geographic isolation to sociodemographic variables, as potential determinants contributing to the widespread practice of SM. However, prior research within this country has not thoroughly examined SM through a socio-psychological lens, which could encompass societal perceptions regarding medication, access to healthcare services, and subsequent care-seeking behaviors among individuals who engage in SM [4].

It is increasingly evident that there is a growing availability of over-the-counter (OTC) medications accessible to the public. The practice of SM with improper usage of drugs could easily result in a variety of complications. The most common and noteworthy among those are adverse drug reactions (ADRs) due to the interactions between various drugs that have been consumed. Many researches have indicated that taking multiple medicines increases the probability of experiencing ADRs. In addition, this risk is further increased in old age as numerous studies have indicated that the consumption of non-prescription drugs could exceed that of prescribed drugs by up to seven times [5]. Hepatic and renal issues, increased fat levels, and exacerbation of pre-existing health conditions could be the most common challenges faced by patients of the elderly age group. Notably, approximately one-third of adult hospital admissions are due to the misuse of non-prescription drugs. Furthermore, the elevated frequency of drug intake increases the risk of experiencing side effects. These side effects encompass various issues, including drug interactions, anaphylactic reactions, impairment of drug tolerance, and the masking of underlying clinical symptoms indicative of serious conditions. Therefore, there is a pressing need for concerted efforts to address this issue and enhance public awareness regarding the potential adverse effects of SM [5].

Nonetheless, SM is not always considered a negative behavior. SM using OTC medications is regarded as a crucial element of self-care. It appears that when the advantages of SM are mentioned, the drugs that a person is permitted to take without having to see a doctor are discussed [6]. The World Health Organization (WHO) states that these products have been produced with strict guidelines and have unique packaging that comes with patient information, all of which can be harmful if ignored [7]. The significance of SM has piqued the interest of healthcare professionals, including physicians and policymakers, particularly as medications transition from prescription-only to OTC availability. The OTC medications and the ways in which they are provided vary by country, and the health systems of each country determine how easy it is to obtain and how often people use them [8].

Self-care has been practical long prior to formal health systems, and it also impacts health outcomes [9]. Since the inception of the primary healthcare movement [10], self-care initiatives in the health sector have increased, fueled by a focus on women's empowerment and the role of self-care in the management of mental health [11] and chronic diseases. Generally, SM is acknowledged to play a vital role in managing minor illnesses. New self-care configurations are becoming feasible because of the rapidly advancing digital technology and expanding global market. While many of these interventions will be subject to regulations, many others will not. This aspect of self-care was underscored by the WHO in 1978 as part of its "Health for All by the Year 2000" initiative, which was adopted in numerous countries worldwide, including Saudi Arabia. Increased prevalence of SM among the general population has been associated with various potential benefits and risks.

Research indicates that the decision to self-medicate is influenced by a multitude of personal, organizational, and environmental factors [12]. Self-care gained attention and notoriety during the COVID-19 pandemic when people started practicing hand hygiene, wearing masks, and performing self-testing at home [13]. SM is a practice observed across all age groups, but a particular focus is directed toward the elderly concerning treatment and medication issues. Numerous studies have highlighted the elderly as the predominant medicine consumers in many countries, a trend also observed in Iran. This heightened medication consumption among the elderly can be attributed to several factors, including the increased prevalence of various diseases associated with aging. Conditions, such as diabetes, cardiovascular disease, and cancer, are prevalent among a significant portion of the elderly population. Additionally, the presence of multiple chronic diseases concurrently, known as comorbidity, is widespread in this age group and often necessitates increased medication usage [7]. SM has long been a prevalent practice within the general populace. This phenomenon is driven by various factors, including the desire for self-care, empathy toward unwell family members, limited access to healthcare services, economic constraints, lack of awareness, misconceptions, widespread advertising, and the availability of medications outside of traditional medical outlets. Additionally, the literature suggests that reasons for engaging in SM include perceptions of illness severity, past experiences with similar illnesses, financial considerations, and difficulties accessing healthcare professionals [14,15]. SM practices should not be universally deemed harmful. OTC medications, available without a prescription, often offer convenience and cost savings for patients. Particularly in remote areas such as hill regions, tribal communities, and other underserved areas with limited access to healthcare professionals, SM remains a crucial recourse for addressing minor symptoms due to the scarcity of medical personnel [16]. Few studies have been conducted at a community level in India to evaluate the prevalence of SM practices. Such studies hold the potential to offer valuable insights into the motivations behind patient’s adoption of this practice. Moreover, they could aid policymakers and regulatory bodies in refining drug regulations, revising the list of essential medicines, and addressing safety concerns related to OTC medications. With this background, the study protocol aims to estimate the prevalence of SM of prescription drugs with their OTC access and the risk practice and identify potential factors that could influence SM practices in pharmacy outlets of Wardha along with reviewing policies pertaining to OTC and prescription drugs and identifying improvements to mitigate the risk practices.

## Materials and methods

Study design, participant, and setting

A convergent parallel mixed method study design with a quantitative component as a cross-sectional study and a qualitative component as an in-depth interview will be used for data collection for a period of 24 months from urban communities and pharmacies of Wardha. Before data collection, informed consent from study participants will be obtained, clearly outlining the purpose of the study, benefits and potential risks, voluntary participation, and the right to withdraw at any time. Ensuring participant confidentiality and privacy while collecting data is essential, and any ethical considerations or limitations in data collection will be carefully addressed to uphold research ethics standards. The study sought approval on 01.03.2024 from the Institutional Ethics Committee of Datta Meghe Institute of Higher Education and Research with reference number DMIHER(DU)/IEC/2024/126 to ensure the research adheres to the ethical guidelines, respects participant rights, and meets established ethical standards and has been registered for the Clinical Trials Registry - India with the registration number CTRI/2024/07/069886.

Inclusion and exclusion criteria

Subjects over 18 years and above who reside in Wardha and visited the pharmacy outlets to purchase medicine(s) during the study will be included in the study. Individuals participating willingly, voluntarily, and providing informed consent, who have a history of SM or are currently engaging in SM practices, and who have the cognitive ability to understand and respond to questions and participate in qualitative interviews. Doctors and healthcare workers who have knowledge of medications, individuals with cognitive impairments, and pregnant or breastfeeding women will be excluded from the study.

Sample size

The sample was calculated based on,

n = [Z2 P (1-P)]/d2

Z is the statistic corresponding to the level of confidence,

P is the expected prevalence,

d is precision (corresponding to effect size).

Sample size calculation

Assumptions:

Precision = 5.0% (0.05)

Prevalence = 11.9% (0.10) [16]

Z = 1.96

N = 1.96*1.96*0.119 (0.881)/0.0025

The final sample size based on the prevalence of 11.9% was 161. Assuming 10.0%-12.0% dropouts, the estimated sample taken was 180.

Data collection tools and methods

Data for this study will be gathered through in-depth semi-structured interviews or questionnaires of 15 to 20 pharmacists and the general population during the study period. These interviews will be administered by the primary researcher, a resident trained in qualitative interview techniques. Additionally, available documents and evidence, like lists of OTC medicines, relevant regulations, prescription records, participants' medication bags, and their home medicine inventories, will be utilized. Each interview will range from 40 to 90 minutes, depending on the respondent's willingness and circumstances. To ensure comprehensive coverage, participants will be given an opportunity to informally converse at the conclusion of the interview, with a prompt such as "Would you like to share anything else?" Subsequent interviews may be scheduled if participants wish to provide further information or if the initial analysis suggests the need for additional data Figure 1. The questionnaire will include Table 1.

**Figure 1:**
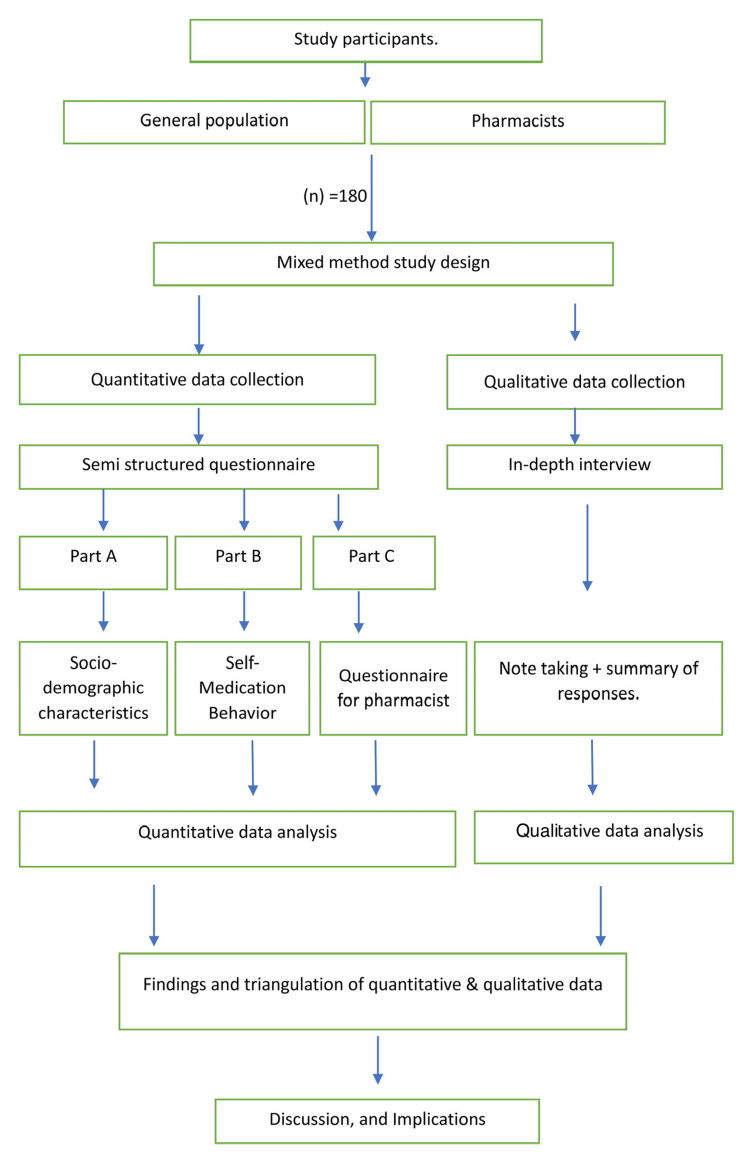
Schematic flowchart for mixed method study design

**Table 1:** Description of the tool included in this study

Section	Details
Part A	Sociodemographic characteristics
	Age
	Place of residence
	Religion
	Marital status
	Level of education
	Occupation
	Family type
	Socioeconomic status (evaluated using Modified B.G. Prasad Scale)
Part B	Self-medication behaviors in the past year
	Patterns of self-medication (e.g., frequency, duration)
	Types of medications used
	Reasons for self-medication (e.g., convenience, cost, perceived need)
Part C	Pharmacist's perspective on self-medication practices
	Inquiry by a doctor to pharmacist
	Pharmacist's observations
	Customer behavior related to self-medication

Data analysis

All collected data will be analyzed using Statistical Package for the Social Sciences (IBM SPSS Statistics for Windows, IBM Corp., Version 23, Armonk, NY). Quantitative data will be presented as mean ± standard deviation, while qualitative data will be expressed as frequencies and percentages. Descriptive statistics will summarize and present sociodemographic characteristics, SM frequencies, patterns, and types of medications used. Measures, such as mean, median, and standard deviation, will be utilized to describe central tendency and variability. Inferential statistics will be employed to analyze relationships and associations between variables. Correlation analysis may explore the degree of association between factors such as age, socioeconomic status, and SM frequency. Furthermore, the chi-square test will assess the impact of demographic variables on SM practices. Group comparisons will be conducted using the student t-test. A p-value <0.05 will be considered statistically significant.

## Results

The study is expected to provide insights into the prevalence of SM practices, explore the factors influencing it, elucidate the reasons driving individuals to self-medicate, and delve into experiences and perceptions to shed light on self-care behaviors in Wardha. It will help in providing recommendations for interventions to promote responsible self-care practices. These outcomes will not only inform strategies to enhance patient education on safe SM and improve access to healthcare services but also have policy implications for public health initiatives aimed at safeguarding individuals from the potential risks associated with SM while empowering them to make informed decisions about their health.

## Discussion

Many factors can lead people to take their own medication, including the need for self-care, empathy for sick family members, a lack of health services, poverty, misinformation, misbelief, and the accessibility of medications in places other than pharmacies. Research conducted in two phases in India has indicated the prevalence of SM among students to be 74.6% and 69.4% [17]. Similar studies suggest that literacy level, history of SM, profession, unavailability of medical insurance, lack of priority in consulting medical professionals, low economic status, the female sex, and staying in an urban setting were among the factors associated with SM practices [17]. Given the growing public health concerns around SM with prescription drugs and OTC access, this study focuses on risk practice prevalence, its use, its safety, and reasons for using it. On one side, the availability of medicines on an OTC basis improves access to healthcare for people living in settings lacking in resources or in places in need of adequate healthcare infrastructure. On the other, it increases the probability of severely harming one's health by the misuse of medications [7]. The dichotomy needs thorough investigation, allowing us to balance accessibility and responsible drug consumption. It is essential for individuals using medications to have appropriate medical comprehension regarding dosage, timing of administration, and potential ramifications of overuse, as inadequate information could lead to major repercussions, including antibiotic resistance, skin issues, hypersensitivity, allergies, and dependency. As it stands, regulations around the OTC availability of certain prescription drugs vary globally across jurisdictions. There is an urgent need to enhance awareness and enact regulations aimed at promoting prudent and safe practices regarding SM. There is a potential to encourage its rational use and mitigate the emergence of microbial resistance by fostering and improving its knowledge. Despite existing research on this subject, SM has slipped under the radar regarding receiving the attention it warrants as a research area. Moreover, most studies on this topic have been conducted in countries with vastly different healthcare systems and cultural contexts from that of India, rendering their findings potentially non-transferable. It is imperative to gather data on the prevalence of SM in India as well as explore their related factors to facilitate the enhancement of SM practices within the country.

Strength

The study focuses on SM practices in Wardha, encompassing diverse demographics through a cross-sectional design. It offers a comprehensive understanding by examining multiple factors influencing SM and can help frame interventions targeted toward promoting self-care practices that are holistic and responsible, ultimately resulting in improved healthcare services. This study can potentially influence public health policies and guidelines related to SM practices, highlighting the importance of regulating OTC medication and enhancing patient education.

Limitations

The study’s findings may be limited by sampling bias if the sample population is not representative of the broader adult population in Wardha, potentially impacting the generalizability of the results. The data collection through surveys and subject interviews could be prone to self-reporting due to the participants providing responses that are socially desirable or may inaccurately recall their self-medicating practices. There may also be temporal constraints for long-term trends, variability in policy enforcement effectiveness, and language and cultural factors may also impact the study's findings.

## Conclusions

It is anticipated that the study will shed light on the frequency of SM practices and investigate the factors influencing it. A multifocal perspective of SM practices, aiding in the advancement of interventions to promote safe and effective self-care while mitigating the risks of self-harm through SM, should be prioritized. By scrutinizing the factors influencing SM practices and their prevalence, this study aims to illuminate the balance between self-care and the potential harm associated with unsupervised medication use. The conclusions drawn from the analysis are anticipated to enrich our understanding of the complex interplay of cultural, individual, and societal factors that drive SM practices and drive meaningful change in healthcare delivery, strengthening health systems and safeguarding individuals from potential harm while harnessing the benefits of self-care practices.
